# Serotonin2C Receptors and the Motor Control of Oral Activity

**DOI:** 10.2174/1570159X11311020003

**Published:** 2013-03

**Authors:** Mélanie Lagière, Sylvia Navailles, Marion Bosc, Martin Guthrie, Philippe De Deurwaerdère

**Affiliations:** 1 Université Bordeaux, Institut des Maladies Neurodégénératives, UMR 5293, F-33000 Bordeaux, France; 2 CNRS, Institut des Maladies Neurodégénératives, UMR 5293, F-33000 Bordeaux, France

**Keywords:** Serotonin2c receptor; oral activity; neuroleptic; 6-hydroxydopamine lesion; hypersensitized oral responses.

## Abstract

Data from many experiments has shown that serotonin2C (5-HT_2C_) receptor plays a role in the control of orofacial activity in rodents. Purposeless oral movements can be elicited either by agonists or inverse agonists implying a tight control exerted by the receptor upon oral activity. The effects of agonists has been related to an action of these drugs in the subthalamic nucleus and the striatum, the two input structures for cortical efferents to the basal ganglia, a group of subcortical structures involved in the control of motor behaviors. The oral effects of agonists are dramatically enhanced in case of chronic blockade of central dopaminergic transmission induced by neuroleptics or massive destruction of dopamine neurons. The mechanisms involved in the hypersensitized oral responses to 5-HT_2C_ agonists are not clear and deserve additional studies. Indeed, while the oral behavior triggered by 5-HT_2C_ drugs would barely correspond to the dyskinesia observed in humans, the clinical data have consistently postulated that 5-HT_2C_ receptors could be involved in these aberrant motor manifestations.

## INTRODUCTION

The serotonin2C (5-HT_2C_) receptor, one of seven transmembrane G-protein coupled receptor in the 5-HT family, is widely expressed in the central nervous system [1, 2], where it plays a major role in the regulation of neuronal network excitability [3]. Its function is multifaceted as it operates through three distinct modalities, i.e. phasic, tonic (involving the spontaneous release of 5-HT) and constitutive activity [4], a receptor activity occurring in the absence of endogenous 5-HT or other agonists and abolished by inverse agonists [5]. Clinical and preclinical research has highlighted its involvement in various brain diseases, leading to the idea that 5-HT_2C_ receptors would possibly make a good target for treating some neuropsychiatric disorders [6-9].

Dyskinesia is a side effect of current therapies for psychosis in schizophrenia and motor impairment in Parkinson’s disease. However antipsychotics are generally dopaminergic (DA) antagonists and parkinsonian medications are DA agonists. Despite the opposite nature of the treatments used in these pathologies, clinical evidence suggests the participation of 5-HT_2C_ receptors in the motor side effects elicited by both the DA agonists and antagonists [10-12]. The occurrence of dyskinesia in the orofacial sphere is supported by preclinical studies in rodents showing that 5-HT_2C_ receptor stimulation or blockade promotes abnormal orofacial and purposeless oral responses. Classically, 5-HT_2C_ agonists have been shown to inhibit DA neuron activity and DA-triggered behaviors [13, 14]. This is in contrast with the oral dyskinesia induced by DA agonists for which 5-HT2C receptors play a permissive role and their impact is dramatically enhanced in the case of impaired DA transmission.. 

The purpose of this review is to stress that 5-HT_2C_ receptors exert a tight control of orofacial activity. After recalling briefly the nature of oral bouts and their relation to human pathophysiology, we will present pharmacological evidence demonstrating that alterations of phasic and constitutive controls of 5-HT_2C_ receptors may promote abnormal and purposeless oral movements. We will focus on the basal ganglia, a group of subcortical structures involved in the control of motor behaviors [15], which constitute an important target for the interaction of 5-HT_2C_ ligands with DA transmission. Thereafter, we will highlight the outcome of 5-HT_2C_ receptor-dependent oral responses in a preclinical model of tardive dyskinesia and Parkinson’s disease.

### I 5-HT_2C_ Receptors and Orofacial Movements

**Fig. (1):**
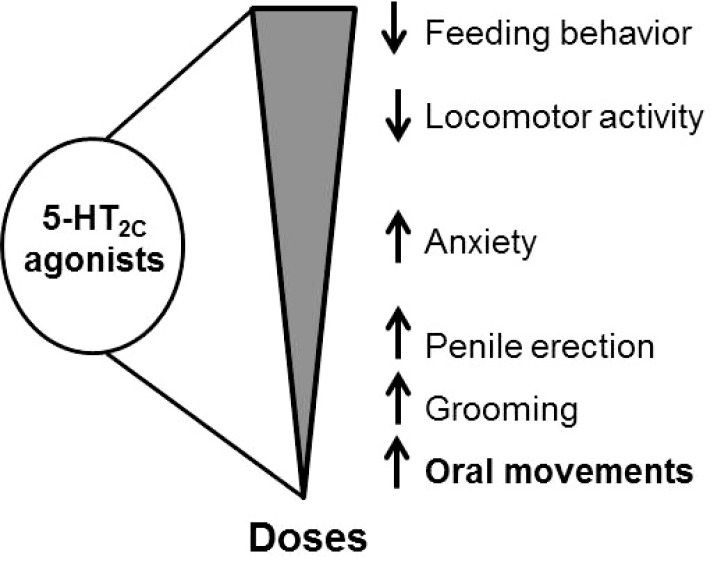
5-HT_2C_ agonists elicit a variety a behavioral manifestations. Their appearance depends on the dose of the agonist used. Purposeless oral movements occur at low doses of non selective and selective 5-HT_2C_ agonists.

The available, non-selective, 5-HT_2C_ agonists elicit various alterations of behavioral responses including grooming, penile erection, hypolocomotor activity, decrease in feeding behavior, anxiety, and purposeless oral movements [16]. These alterations appear as a function of the doses of agonists administered, the decrease in feeding behavior and locomotor activity occurring at higher dosage (approximately 1 mg/kg) compared to the other responses (Fig. **1**). The purposeless oral behavior consists of vacuous chewing, jaw tremor, and tongue darting occurring without any physical purpose and is elicited at low doses of agonists (0.1-0.3 mg/kg). This hyperkinetic syndrome of repetitive oro-buccal movements has been shown to be generated by chronic treatment with neuroleptics and has been used as a rodent model of tardive dyskinesia [17]. Similarly, abnormal orofacial movements are used to score antiparksionian drug-induced dyskinesia in rodent model of Parkinson’s disease [18]. In other situations, the occurrence of drug-induced tremulous jaw movements has been related to resting tremor observed in Parkinson’s disease based on the frequency of these movements [19]. 

The notion that abnormal and purposeless oral movements elicited by various drugs in rodents may model a specific human pathology is not totally clear. Individual bouts of oral movements (single movement of jaws, or mouth) can occur occasionally in naïve rodents. Therefore these movements correspond, in the case of drug administration, to an exaggeration of the normal animal’s behavioral repertoire. Furthermore, behavior is triggered by 5-HT compounds in rodents [20] but not in primates [21, 22]. The behavioral response elicited by 5-HT and DA agents in rodents includes associative/limbic territories of the basal ganglia [23], is extremely sensitive to arousal, and as noted above, occurs with complex pattern of oral response and is associated with grooming [20, 24]. Thus, in addition to neuroleptic-induced tardive dyskinesia, the complex oral responses elicited by 5-HT and DA drugs could correspond to tics, compulsive behavior or Tourette’s syndrome. Moreover, grooming elicited by 5-HT_2C_ receptor stimulation, although not considered as “purposeless oral movements”, has been also proposed to mimic some aspects of obsessive compulsive disorder [25], another neuropsychiatric disease thought to come from aberrant signalling in associative/limbic part of basal ganglia. Polymorphisms in the 5-HT_2C_ gene have been reported in neuroleptic-induced dyskinesia and extrapyramidal side effects [26, 27] and also in Tourette’s syndrome [28]. Thus, while the parallel toward a specific human pathology is not established, the purposeless oral responses elicited by 5-HT drugs in rodents can be studied to further the human and rodent data strongly suggesting a role for 5-HT, and presumably 5-HT_2C_ receptors, in oral motor control.

#### Stimulation of 5-HT_2C_ Receptors Increases Purposeless Oral Behavior

Stewart *et al*. [20] observed an increase in purposeless oral movements elicited by the non-selective 5-HT agonists *m*-CPP, trifluoromethylphenylpiperazine (TFMPP) and quipazine. The intensity of oral bouts induced by the 5-HT agonists is dose-dependent [20, 29, 30]. In general, the magnitude of the oral responses is smaller compared to DA or cholinergic agonists [19, 30, 31]. Although *m*-CPP may bind to several 5-HT receptors and the 5-HT transporter [32], extensive pharmacological characterization indicates that the oral bouts induced by *m*-CPP rely on 5-HT_2C_ receptor-dependent mechanisms. A variety of 5-HT_2C_ receptor blocking agents, including mianserin, mesulergine, SDZ SER 082, SB 206553 or SB 243213 can suppress these *m*-CPP-induced oral responses [20, 29-31, 33, 34]. Conversely, 5-HT_1B_, 5-HT_2A_ or 5-HT_3_ antagonists did not modify *m*-CPP-induced abnormal oral movements while 5-HT_1A_, 5-HT_1B_ or 5-HT_3_ agonists did not elicit oral dyskinesia [20, 30, 35]. Oral movements observed after systemic injection of *m*-CPP were not modified or transiently increased by 5,7-dihydroxytryptamine lesions of 5-HT neurons [36], thus ruling out the action of *m*-CPP on the 5-HT transporter in this behavioral response. Although these latter data do not exclude a role for 5-HT receptors other than 5-HT_2C_ ones in the control of orofacial activity, these results emphasize a strong link between oral motor control and 5-HT_2C_ receptors.

**Fig. (2):**
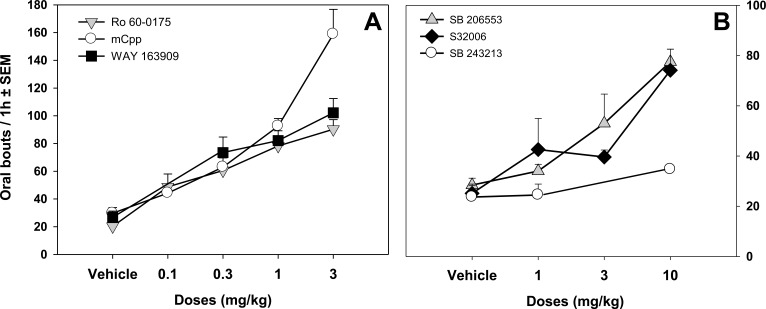
**5-HT_2C_ receptor agonists, inverse agonists, but not antagonist, elicit oral bouts. A.** Data represent the mean ± SEM of oral bouts recorded for 1 hour (1h) following the intraperitoneal administration of non selective, preferential and selective 5-HT_2C_ agonists : *m*-CPP, Ro 60-0175 and WAY 163909 respectively or their vehicle. (n = 4-7 rats/group). The lowest injected dose of Ro 60-0175 (0.1 mg/kg) already induced a significant increase in oral movements. The dose of 0.3 mg/kg of WAY 163909 or *m*-CPP significantly enhanced oral bouts. **B**. Data represent the mean ± SEM of oral bouts recorded for 1 hour (1h) following the intraperitoneal administration of the antagonist SB 243213 (1 and 10 mg/kg) or the inverse agonists, SB 206553 (1, 3 and 10 mg/kg) and S32006 (1, 3 and 10 mg/kg). The administration of inverse agonists dose-dependently increased oral bouts in naïve rats. SB 206553 increases oral movements at the dose of 3 and 10 mg/kg whereas the S32006 effects reached significance only at the maximal dose (10 mg/kg). The antagonist SB 243213 did not affect oral bouts. Adapted from [31].

The use of more selective 5-HT_2C_ agonists has confirmed the link between 5-HT_2C_ receptors and the control of oral behavior. The preferential 5-HT_2C_ agonists Ro 60-0175 and WAY 163909, two piperazine derivatives, were equally potent in promoting oral bouts (Fig. **2A**; Table **1**). The number of oral bouts induced by WAY 163909 was maximal at 3 mg/kg and decreased at higher doses (10 mg/kg) [31], as with *m*-CPP [20]. Compared with other 5-HT_2C_ agonists, WAY 163909 has the highest affinity for 5-HT_2C_ receptors (Ki = 11 nM) and is 20- and 46-fold more selective over 5-HT_2A_ and 5-HT_2B_ receptors, respectively (Table **1**) [37]. WAY 163909 is one of the most selective 5-HT_2C_ agonists available, confirming that the stimulation of 5-HT_2C_ receptors by 5-HT_2C_ agonists promotes purposeless oral movements in rodents. In addition, the bouts of oral movements induced by the 5-HT_2_ agonist Ro 60-0175 were abolished by the selective 5-HT_2C_ antagonist SB 243213 [31, 38]. 

#### Blockade of 5-HT_2C_ Receptor Function may Promote Abnormal Oral Activity

Most of the available data indicate that both non-selective and selective 5-HT_2C_ antagonists, including mianserin, mesulergine, ritanserin and SB 243213, did not alter oral activity by themselves [20, 31, 33, 34, 39]. Low doses of the 5-HT_2C_ antagonists SER082 or SB 206553 were also ineffective [34]. Ritanserin has been reported to elicit vacuous chewing per se in specific conditions [40] but its pharmacological profile, especially with its DA-D2 antagonist properties [41] (see below), preclude accurate interpretation. On the other hand, recent data also showed that higher doses of SB 206553 induced a dose-dependent enhancement of purposeless oral movements [31]. The effect was maximal at 10 mg/kg and a higher dose did not increase further the magnitude of oral bouts. Similarly, the 5-HT_2C_ antagonist S32006 dose-dependently increased oral bouts [31, 42] (Fig. **2B**). 

SB 206553 is a prototypical 5-HT_2C_ inverse agonist *in vitro* while S32006 behaves as a partial 5-HT_2C_ inverse agonist [43]. Several studies have reported that SB 206553 can behave as an inverse agonist *in vivo*, generating different responses including head bobs in rabbits [44], functional motor recovery after lesion of the spinal chord [45] or the control of subcortical DA release [46]. Although the 5-HT_2B_ component of SB 206553 and S32006 could not be excluded (Table **1**), the effects of these compounds on oral behavior have been related to their ability to block the constitutive activity of native 5-HT_2C_ receptors due to their inverse agonist properties. Indeed, SB 243213 fully abolished the oral bouts induced by increasing doses of SB 206553. Furthermore, the increase in oral bouts induced by S32006 was blocked by SB 243213 and was unaffected by the selective lesion of 5-HT neurons induced by intra-raphe injection of 5,7-dihydroxytryptamine [31]. Of note, the 5-HT lesion per se does not induce abnormal movements [30, 31, 36] suggesting that the removal of the 5-HT tone is not sufficient to unmask 5-HT_2C_ receptor-dependent control of oral motor activity. Moreover, although other compounds such as mesulergine or ritanserin can display inverse agonist properties, their non selective profile may mask such a property [47, 48]. Thus, the orofacial effects produced by the pharmacological inhibition of 5-HT_2C_ receptor function were reminiscent of the increased chewing activity reported in 5-HT_2C_ receptors knock-out mice compared to wild-type [49]. 

Overall, these data suggest that orofacial activity is under a tight control exerted by different activities of the 5-HT_2C_ receptor, including the constitutive activity of native 5-HT_2C_ receptors.

### II The Orofacial Effects of 5-HT_2C_ Agonists Involve the Basal Ganglia

5-HT_2C_ receptors are widely expressed in the central nervous system [2, 50]. The link between the enhanced oral activity induced by 5-HT_2C_ receptor stimulation and the basal ganglia is related to the presence of the receptor in these brain regions, to direct evidence using local administration of agonists, and, more generally, to the known involvement of the basal ganglia in extrapyramidal side effects induced by DA therapy. An additional action of 5-HT_2C_ ligands in medulla or spinal chord has not been excluded [51] but no data are available to further this hypothesis. 

A comparison between mRNA and binding sites suggests that the 5-HT_2C_ receptor is mostly a somatodendritic receptor, except in the external globus pallidus (GPe), where it may be located on axons [52-54]. Numerous cell types express the receptor including GABAergic, glutamatergic, and cholinergic neurons [55]. DA neurons may express very low levels of 5-HT_2C_ receptors in the substantia nigra pars compacta (SNc) but greater levels are found in the ventral tegmental area (VTA) [13, 52]. In general, the density of 5-HT_2C_ receptors follows the density of the 5-HT innervation, the ventromedial parts of basal ganglia being enriched in both. 

**Fig. (3):**
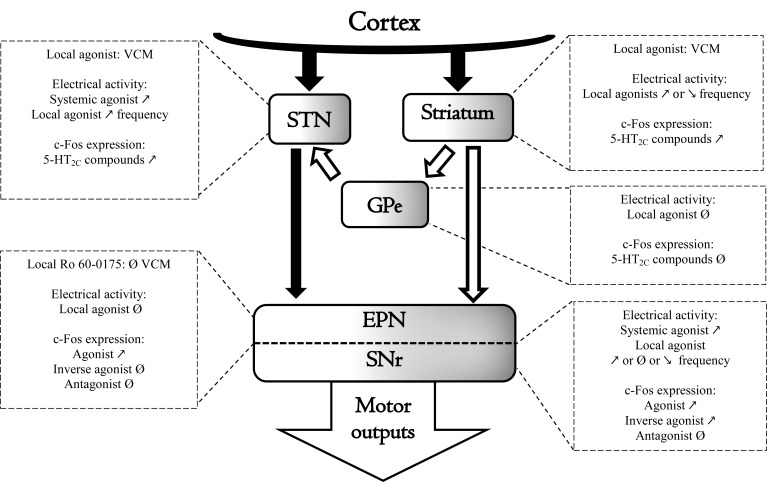
Schematic summary of 5-HT_2C_ receptor impact on basal ganglia motor control (VCM : Vacuous Chewing Movement) and functional anatomic datas of 5-HT_2C_ receptor in the basal ganglia nuclei. ↗ or ↘ indicate respectively an increase or a decrease of the studied parameter whereas Ø indicate a lack of effect of 5-HT_2C_ compounds. Black arrows indicate excitatory projections and white arrows are representing inhibitory projections between the basal ganglia nuclei subthalamic nucleus (STN); striatum; external globus pallidum (GPe); entopeduncular nucleus (EPN); substantia nigra pars reticulata (SNr) and the cortices and motor output structures. The shadow part of the nuclei represent the ‘associative/limbic’ territories of basal ganglia, in which the expression of 5-HT_2C_ receptor is more important.

Intracerebral microinjections of *m*-CPP have demonstrated that the abnormal oral responses involved an action of the drug at receptors located within the basal ganglia, specifically in the subthalamic nucleus (STN) and the striatum (Fig. **3**). Indeed, either unilateral or bilateral administration of low doses of *m*-CPP into the STN elicits oral bouts, an effect that can be blocked by mesulergine [33, 56]. Furthermore, the oral bouts elicited by the systemic administration of *m*-CPP are abolished by the intra-STN administration of mesulergine [33]. While the above studies favour the idea of an almost exclusive influence of STN 5-HT_2C_ receptor in the effects of *m*-CPP, Plech *et al*. [57] have also reported that intrastriatal administration of *m*-CPP induced purposeless oral movements that were abolished by the intrastriatal administration of mianserin. Interestingly, despite the role of the entopeduncular nucleus (EPN; the equivalent of the internal globus pallidus in primates) in mediating abnormal movements [58], stimulation of EPN 5-HT_2C_ receptors by the local administration of *m*-CPP did not stimulate bouts of oral movements [59]. On the other hand, high doses of *m*-CPP or TFMPP directly administered into the substantia nigra pars reticulata (SNr) have been shown to elicit abnormal oral movements [60]. For these authors, the fact that the non-selective 5-HT_2_ agonist DOB did not induce vacuous chewing suggests a role for 5-HT_1B_ receptors in the effects triggered by intra-SNr *m*-CPP. Altogether these data suggest that striatal and STN 5-HT_2C_ receptors are specifically involved in the abnormal oral responses induced by 5-HT_2C_ agonists in naïve rats. 

The neurobiological data validate the idea that 5-HT_2C_ receptors located in the basal ganglia may be responsible for the oral movements induced by 5-HT_2C_ agonists (Fig. **3**). Stimulation of 5-HT_2C_ receptors may enhance the activity of STN or SNr cells *in vitro* and *in vivo* [61-65]. Electrophysiological changes have also been reported in the striatum [51, 52] but not in the GPe or the EPN [66, 67]. The administration of non-selective 5-HT_2C_ agonists enhanced the protooncogene c-Fos, a marker of changes of neuronal activity in the striatum and the STN [38, 56, 68, 69]. The role of 5-HT_2C_ receptors in these effects would be only partial because selective 5-HT_2C_ antagonists did not fully block the induction of c-Fos induced by non-selective 5-HT_2C_ agonists (Navailles *et al*., unpublished observation). Similarly, selective antagonists and inverse agonists may also induce c-fos expression in the STN and the striatum without altering the frequency of discharge of STN or SNr neurons *in vivo *[4, 62, 70-72].

As a general comment, the pattern of expression of the 5-HT_2C_ receptor suggests that the functional influences of the 5-HT_2C_ receptor may be stronger on associative and limbic circuits than on the sensorimotor circuits. Some [38, 56, 68, 69], but not all [73, 74] studies have suggested a preferential action of 5-HT agonists toward associative and limbic territories of the basal ganglia. 

In the basal ganglia, 5-HT_2C_ receptors could interact with DA and cholinergic transmission, both of which are known to alter oral motor responses [19], but the data are controversial. Thus, non selective 5-HT_2C_ receptor antagonists reduce oral bouts elicited by DA and muscarinic agonists [30, 75]. Rosengarten *et al*. [76] have shown that the effect of DA agonists and *m*-CPP on purposeless oral movements are additive, suggesting that these compounds may operate *via *distinct mechanisms. Nonetheless, mianserin is able to slightly reduce abnormal orofacial movements induced by DA agonists [30, 76] and especially D1 agonists [77]. In another study, mianserin blocked tacrine-induced vacuous chewing when injected into the dorsolateral part of SNr [75], a zone involved in facial motor control [78]. The role of 5-HT_2C_ receptors in the latter response deserves caution considering that mianserin is not selective for 5-HT_2C_ receptors and that selective 5-HT_2C_ antagonist SB 243213 did not reduce the bouts of oral movements stimulated by the muscarinic agonist pilocarpine [31]. Creed *et al*. [79] showed that the duration of tremor induced by the acetylcholine esterase inhibitor physostigmine, a behavioral alteration that is gradually transferred from the head to the entire body, was reduced by a high dose of ritanserin while the 5-HT synthesis inhibitor parachlorophenylalanine prevented physostigmine-induced tremor. Moreover, a non-selective 5-HT agonist 5-methoxy-N,N-dimethyltryptamine induced tremor per se. The role of 5-HT in tremor is interesting in the context of Parkinson’s disease as resting tremor could be related to aberrant signalling of the 5-HT system [80, 81]. However, the above behavioral data obtained in rodents does not favour the specific participation of 5-HT_2C_ receptors in this clinical sign. 

### III Neuroleptic-Induced Dyskinesia and the Increase of 5-HT_2C_ Receptor Function

The link between the 5-HT_2C_ receptor and the control of orofacial movement is of particular importance with regards to the chronic use of antipsychotics [8]. Classical antipsychotics such as haloperidol cause motor side effects named tardive dyskinesias, which are classified as a neurological syndrome and characterized by repetitive involuntary, purposeless (i.e. vacuous) chewing movements with or without tongue protrusion and lip smacking [82, 83]. Tardive dyskinesia is a movement disorder that develops gradually and usually only after long-term treatment with an antipsychotic. To this end, Waddington *et al*. [84, 85] first showed that long-term treatment with neuroleptics resulted in spontaneous orofacial dyskinesias in the rat [8]. The precise mechanisms underlying vacuous chewing movements after long-term antipsychotic treatment are likely related to the primary mechanism of action of all antipsychotic drugs, namely the blockade of DA-D2 receptors [86]. The 5-HT system and notably 5-HT_2C_ receptors appeared to be also involved in the production of these debilitating motor side effects [8]. This implication is further established in humans as the propensity of neuroleptic-induced dyskinesia is correlated to polymorphisms of the 5-HT_2C_ receptor [87]. In 2008, Richtand [88] suggested that the interaction between DA-D2 and 5-HT_2C_ receptors may participate in the therapeutic response achieved following treatment with typical antipsychotic medications [88]. However, the exact role of 5-HT_2C_ receptors in this phenomenon remains to be elucidated. 

The data in rodents supports a role for the 5-HT system and 5-HT_2C_ receptors in the oral response consequent to chronic administration of neuroleptics. Thus, chronic treatment with haloperidol increases oral responses induced by *m*-CPP [34, 89]. Furthermore, the dyskinesia measured after weeks of treatment with haloperidol is reduced by the non-selective 5-HT_2_ antagonists ritanserin, seganserin, or ketanserin [40]. Haloperidol-induced oral dyskinesia can be reduced by concomitant daily administration of ritanserin, and the dyskinesias persisting after haloperidol withdrawal can also be reduced by ritanserin administration [39]. More recently, it has been reported that 5-HT_2C_ but not 5-HT_2A_ antagonists attenuated chronic haloperidol induced vacuous chewing movements. These behavioral effects were paralleled by changes in 5-HT_2C_ but not 5-HT_2A_ mRNA levels in several brain regions including medial caudate-putamen after chronic haloperidol treatment [90]. The finding that the 5-HT_1A_ agonists 8-OH-DPAT and buspirone also reduced oral dyskinesia induced by long-term haloperidol treatment suggests that 5-HT tone is altered by this neuroleptic [91, 92]. Indeed, haloperidol can enhance classical responses that are dependent on somatodendritic 5-HT_1A_ receptors such as the control of 5-HT metabolism or locomotor responses altered by 8-OH-DPAT [93]. Chronically co-administering buspirone with haloperidol progressively suppresses haloperidol-induced oral dyskinesias. However, Wolf *et al*. [34] reported a slight increase in *m*-CPP-induced inositol phosphate accumulation in striatal tissue of rats chronically treated with haloperidol. Moreover, Ikram *et al*. [89] have shown a greater increase in 5-HT metabolite tissue content induced by *m*-CPP in the dorsolateral striatum of chronically haloperidol-treated rats. Altogether, these data show that haloperidol alters central 5-HT neurotransmission, possibly by changing both the activity of 5-HT neurons, leading to alteration in 5-HT release, and the responses of 5-HT_2C_ receptors, by modifying the coupling efficiency of the receptor with intracellular second messenger pathways. 

There are limitations to these conclusions. First, most of the above-mentioned data in rodents have been obtained with 5-HT drugs that are not selective for 5-HT_2_ receptors and some of them (buspirone, ritanserin) display affinities for DA-D_2_ receptors [41, 92]. The contribution of this pharmacological component cannot be excluded from the interpretation of their antidyskinetic properties. Second, to the best of our knowledge no explicit data are available regarding the responses to 5-HT in other basal ganglia regions in animals chronically treated with haloperidol. These studies would be important to provide a comprehensive explanation of the greater responsiveness to 5-HT_2C_ agonists under these conditions. The STN could be an important locus. Indeed, high frequency stimulation (HFS) or lesions of STN has been shown to significantly alleviate oral dyskinesias in rats [79, 94]. STN-HFS in sham-lesioned and DA-lesioned rats has been shown to reduce 5-HT release in several regions, in part *via *the inhibition of 5-HT neuron activity [95]. Similarly, an action in the output regions of the basal ganglia may be envisioned. HFS of the EPN has been shown to reduce oral dyskinesia in rats chronically treated with haloperidol [79]. Moreover, the blockade of 5-HT_2A/2C_ receptors is thought to underlie the inhibitory influence of clozapine and risperidone on the discharge of SNr neurons, and these two antipsychotic drugs have been shown to minimally induce oral dyskinesia in rats and tardive dyskinesia in humans [96]. Indeed, 5-HT_2A/2C_ receptor antagonism as well as moderate DA-D2 receptor antagonism reproduces the *in vivo* effects of these atypical antipsychotics on the firing rate of SNr neurons. These data support the idea that alterations in 5-HT_2C_ receptor neurotransmission may occur in brain areas other than the striatum. Studies in animal models of Parkinson’s disease support this hypothesis (see below).

### IV Orofacial Motor Control and Animal Model of Parkinson’s Disease

Parkinson’s disease has been associated to the progressive destruction of the nigrostriatal DA neurons [97]. The loss of DA induces profound changes in the functional anatomy of the basal ganglia leading to symptoms of parkinsonism, including bradykinesia, rigidity, and tremor at rest [98, 99]. The destruction of DA neurons in animal models of Parkinson’s disease, such as 1-methyl-4-phenyl-1,2,3,6-tetrahydropyridine (MPTP)-treated monkeys, or hemiparkinsonian rats induced by a unilateral injection of 6-hydroxydopamine (6-OHDA), may induce a sprouting of 5-HT fibres in the striatum and the SN, with a corresponding increase in 5-HT tissue content and release in the striatum [83, 84]. In rodents, this increase has been more frequently observed in 6-OHDA rats lesioned as neonates with bilateral lesions, which does not correspond to a model of Parkinson’s disease, compared to 6-OHDA rats lesioned as adults with unilateral lesions [100-102]. Such modifications have not been observed in humans where most of the data would rather support a damage of 5-HT fibres in the brain of parkinsonian patients [81, 103, 104]. The status of the 5-HT system differs between toxin-induced rodent models in which nigrostriatal neurons are selectively destroyed versus Parkinson’s disease, in which multiple neuronal populations can be affected, including the raphe nuclei [81, 105]. Some recent approaches also includes in parkinsonian rodent models 5-HT depletion to take into account the 5-HT component of the human disease [106] while the effect of 5-HT system in the MPTP-treated monkey would depend on the MPTP regimen [7]. Of course, the interpretation in humans is complicated by the presence of treatments and one cannot exclude that the 5-HT damage could have been greater if no sprouting had occurred. Interestingly, the levels of 5-HT_2C_ receptor mRNA do not follow the increase in 5-HT innervation in neonate 6-OHDA rats [107]. Numan *et al*. [108] have shown that adult 6-OHDA lesion of DA neurons did not modify 5-HT_2C_ receptor mRNA in the striatum, at variance with the 5-HT_2A_ receptor mRNA. In humans, 5-HT_2C_ receptor binding is not affected in the brain of parkinsonian patients [109], although an increase in mesulergine binding has been reported specifically in the SNr [110].

**Fig. (4):**
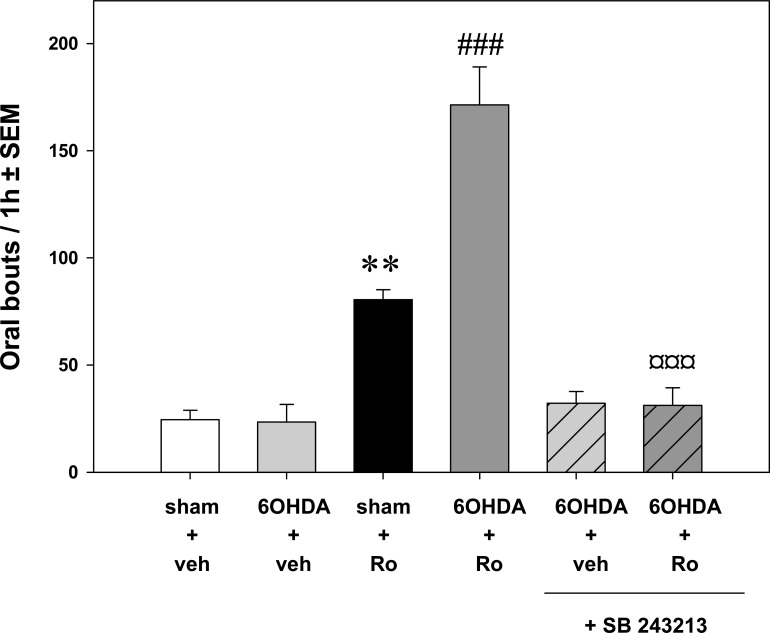
Effect of the nigrostriatal DA lesion on the ability of Ro 60-0175 (3 mg/kg) to stimulate oral bouts. The 5-HT_2C_ agonist or its vehicle (Veh) were administered intraperitoneally (i.p.) in rats receiving an intra-nigral administration of 6-hydroxydopamine (6-OHDA) or its Veh (sham) 3 weeks before. Oral bouts induced by Ro 60-0175 (Ro) are potentiated in 6-OHDA-treated rats. In dopamine-depleted rats SB 243213 does not affect oral movements but blocks the increase in oral bouts induced by Ro 60-0175. Data represent the mean ± SEM of oral bouts recorded for 1 hour following the administration of 5-HT_2C_ agonist or its vehicle (n=4–7 rats/group). ** p<0.01 vs. sham+Veh group; ### p<0.001 vs. sham+Ro group, ¤¤¤ p<0.001 vs. 6-OHDA+Ro group (Fisher’s protected least significance difference test). Adapted from [31].

The data in rodents indicate that nigrostriatal DA lesion enhances the responsiveness to 5-HT agonists. The purposeless oral responses to peripheral injection of *m*-CPP, Ro 60-0175 or WAY 163909 are dramatically enhanced in rats lesioned as adults [31, 56]. The potentiating effect of DA lesion on oral bouts induced by Ro 60-0175 at 3 mg/kg was fully blocked by the selective antagonist SB 243213 (Fig. **4**). Earlier, it had been repeatedly shown that the selective destruction of DA neurons in neonate rats dramatically enhanced the oral effects of peripheral administration of *m*-CPP [29, 30, 111]. Interestingly, neonatal DA neuronal loss also increased the sensitivity of adult rats to oral dyskinesia when challenged with DA drugs [30]. This exaggerated response to DA agonists can be reduced by the non-selective 5-HT_2C_ antagonist mianserin as well as by a lesion of 5-HT neurons with 5,7-dihyroxytryptamine [77]. Nigrostriatal DA lesions in adults enhanced orofacial responses induced by agonists but not by the 5-HT_2C_ inverse agonist SB 206553. Thus it can be concluded that the hypersensitivity of the oral responses to 5-HT_2C_ agonists in DA-lesioned rats involves 5-HT_2C_ receptor-dependent controls other than its constitutive activity. It is possible that, the phasic and constitutive influences of 5-HT_2C_ receptors could be related to distinct cell populations [48].

The mechanisms whereby oral responses to 5-HT_2C_ receptor agonism are dramatically enhanced could be related to multiple changes in 5-HT_2C_ receptor mediated neurotransmission in restricted areas of the basal ganglia in DA-lesioned rats [55]. Here, we are considering possible changes at the level of the STN, the striatum and the output regions of the basal ganglia. By examining the expression of the protooncogene c-Fos, we have found that lesions of nigrostriatal DA neurons did not modify the increase in c-Fos expression induced by peripheral administration of *m*-CPP in the STN. Three sets of electrophysiological or behavioral data have confirmed the lack of changes in STN 5-HT_2C_ receptors after a DA lesion. First, the intra-STN administration of low doses of *m*-CPP stimulated oral bouts similarly in both sham- and 6-OHDA-lesioned rats. Second, the ability of *m*-CPP to stimulate the firing rate of STN was similar in naïve versus lesioned rats [72]. Finally, the contraversive turning behavior induced by the intra-STN administration of 5-HT, attributed in part to the stimulation of 5-HT_2C_ receptors, was not affected by lesions of DA neurons [112]. Thus, 5-HT_2C_ receptors of the STN are an important locus to generate oral dyskinesia elicited by *m*-CPP but they are not directly responsible for the greater oral response observed in 6-OHDA-lesioned rats. 

The striatum might be one important locus. Indeed, the c-Fos response induced by *m*-CPP is decreased in the medial, but not the lateral striatum of 6-OHDA-lesioned rats. This data is difficult to interpret due to the multiple mechanisms possibly involved in the striatal effects elicited by *m*-CPP [74]. Keeping this in mind, Plech *et al*. [57] have more directly reported that oral dyskinesia induced by the intrastriatal administration of *m*-CPP was increased in 6-OHDA rats lesioned as neonate. Although this effect was blocked by the concomitant intrastriatal administration of mianserin, additional data are needed to confirm a role for 5-HT_2C_ receptors in these responses due to their non-selective profile and the pharmacological biases inherent to the intrastriatal administration of drugs [113]. The behavioral increase could not be related to an increase in 5-HT_2C_ receptor mRNA [107], leading this research group to propose that the modification of 5-HT_2C_ receptor transmission occurred downstream of striatal DA transmission. Together with possible modifications of transduction signalling on striatal cells [34], the most spectacular changes of 5-HT_2C_ neurotransmission in 6-OHDA-lesioned rats occurred in output structures of the basal ganglia. Nigrostriatal DA lesions enhanced the ability of peripheral administration of *m*-CPP to increase c-Fos expression in the EPN on the lesioned side only. Interestingly, the administration of Ro 60-0175 in the EPN of the lesioned side elicited purposeless oral movements [59]. An alteration of 5-HT_2C_ receptor neurotransmission in the basal ganglia output is supported by data showing that intranigral infusions of the 5-HT_2C_ blocking agent SB 206553 elicited contraversive turning behavior when administered to the lesioned side in 6-OHDA-lesioned rats [114]. This locus could be responsible in part for the ability of 5-HT_2C_ receptor antagonists such as normethylclozapine, SB 200646, or SB 206553 to increase the contralateral rotations elicited by the DA-D2 agonist quinpirole or the DA-D1 agonist SKF 82958 in 6-OHDA-lesioned rats [114-116].

## CONCLUSIONS

5-HT_2C_ receptors exert a tight control of oral motor behavior in rodents that involves distinct modalities of function of the receptor and, perhaps, distinct loci. The abnormal orofacial response to 5-HT_2C_ agonists involves, in naïve rats, the two input structures of the system, the STN and the striatum. The functional meaning of the bouts of oral movements triggered by 5-HT_2C_ ligands or other drugs is still difficult to translate to human diseases. Yet, the tight control exerted by 5-HT_2C_ receptors upon oral motor activity in rodents could underscore the association found in humans regarding the occurrence of tardive dyskinesia and other abnormal motor manifestations with some polymorphisms of 5-HT_2C_ receptors [26]. The potentiation of the abnormal oral movements to 5-HT_2C_ receptor stimulation in case of chronic blockade of DA transmission further stresses the need to better understand the neurobiological basis of this behavioral response in rodents.

## Figures and Tables

**Table 1.** Affinity (pKi) of 5-HT Compounds at 5-HT_2A_, 5-HT_2B_ and 5-HT_2C_ Receptors and Intrinsic Activity at 5-HT_2C_ Receptors *in vitro*

Ligand	Type	5-HT_2A_ p*K*_i_±SEM	5-HT_2B_ p*K*_i_±SEM	5-HT_2C_ p*K*_i_±SEM	
**(-)DOI**	Agonist	9.03±0.11	7.55±0.05	8.08±0.11	[117]
***m*-CPP**	Agonist	7.26±0.02	7.39±0.02	7.85±0.07	[117]
**Ro 60-0175**	Agonist	7.44±0.04	8.27±0.06	8.22±0.29	[117]
**WAY 163909**	Agonist	6.67 (*K*_i_=212±29)	6.31 (*K*_i_=485±49)	7.97 (*K*_i_=10.5±1.1)	[37]
**SB 242084**	Antagonist	6.07±0.18	6.84±0.28	8.15±0.10	[117]
**SB 243213**	Antagonist	7.01±0.10	7.20±0.11	9.37±0.09	[118]
**S32006**	Inverse agonist	6.00±0.07	8.03±0.05	8.43±0.06	[42]
**SB 206553**	Inverse agonist	5.64±0.09	7.65±0.07	7.79±0.07	[117]

## ABBREVIATIONS

5-HT_2C_: = serotonin2C receptor receptor
DA: = Dopamine
6-OHDA: = 6-hydroxydopamine
HFS: = high frequency stimulation
STN: = subthalamic nucleus
EPN: = entopeduncular nucleus
GPe: = external globus pallidus
SNc: = substantia nigra pars compacta
SNr: = substantia nigra pars reticulata
VTA: = ventral tegmental area
SB 243213: = 5-methyl-1-[[2-[(2-methyl-3-pyridyl) oxy]-5-pyridyl]carbamoyl]-6-trifluoromethylindoline
SB 206553: = 5-methyl-1-(3-pyridylcarbamoyl)-1,2, 3,5-tetrahydropyrrolo[2,3-f]indole hydrochloride
S32006: = N-pyridin-3-yl-1,2-dihydro-3H-benzo [e]indole-3-carboxamide
Ro 60-0175: = S-2-(6-chloro-5-fluoroindol-1-yl)-1-methylethylamine
WAY 163909: = (7bR,10a*R*)-1,2,3,4,8,9,10,10aoctahydro- 7b*H*-cyclopenta- [b][1,4]diazepino[6,7,1hi]indole
*m*-CPP: = metachlorophenylpiperazine
TFMPP: = Trifluoromethylphenylpiperazine
SER 082: = (+)-cis-4,5,7a,8,9,10,11,11aoctahydro-7H-10-methylindolo[1,7-bc][2,6]- naphthyridine
8-OH-DAT: = 8-hydroxy-2-(di-n-propylamino)tetralin
